# Supplementary material for: Monsoonal imprint on late Quaternary landscapes of the Rub’ al Khali Desert
Source: Commun Earth Environ. 2025 Apr 3;6(1):255. doi: 10.1038/s43247-025-02224-1 (PMC11968403; doi:10.1038/s43247-025-02224-1)
Supplement: Supplementary file 2 — supplementary information [file 43247_2025_2224_MOESM2_ESM.pdf]

Supporting information for

Monsoonal imprint on late Quaternary landscapes of the Rub' al Khali Desert

Abdallah S. Zaki[1,2,3], Antoine Delaunay[4], Guillaume Baby[4], Negar Haghipour[5], Cécile Blanchet[6], Anne Dallmeyer[7], Pietro Sternai[8,9], Sam Woor[10,11], Omar Wani[2,12], Hany Khalil[13], Mathieu Schuster[14], Michael Petraglia[15,16,17], Florence Sylvestre[18], Giovan Peyrotty[1], Mohamed Ali[19], Frans Van Buchem[4], Abdulkader M. Afifi[4], and Sébastien Castelltort[1]

***Corresponding author:** Abdallah S. Zaki (Abdallah.zaki@jsg.utexas.edu)

The PDF file includes:

Figs. S1 to S21

Table legends

Supplementary references

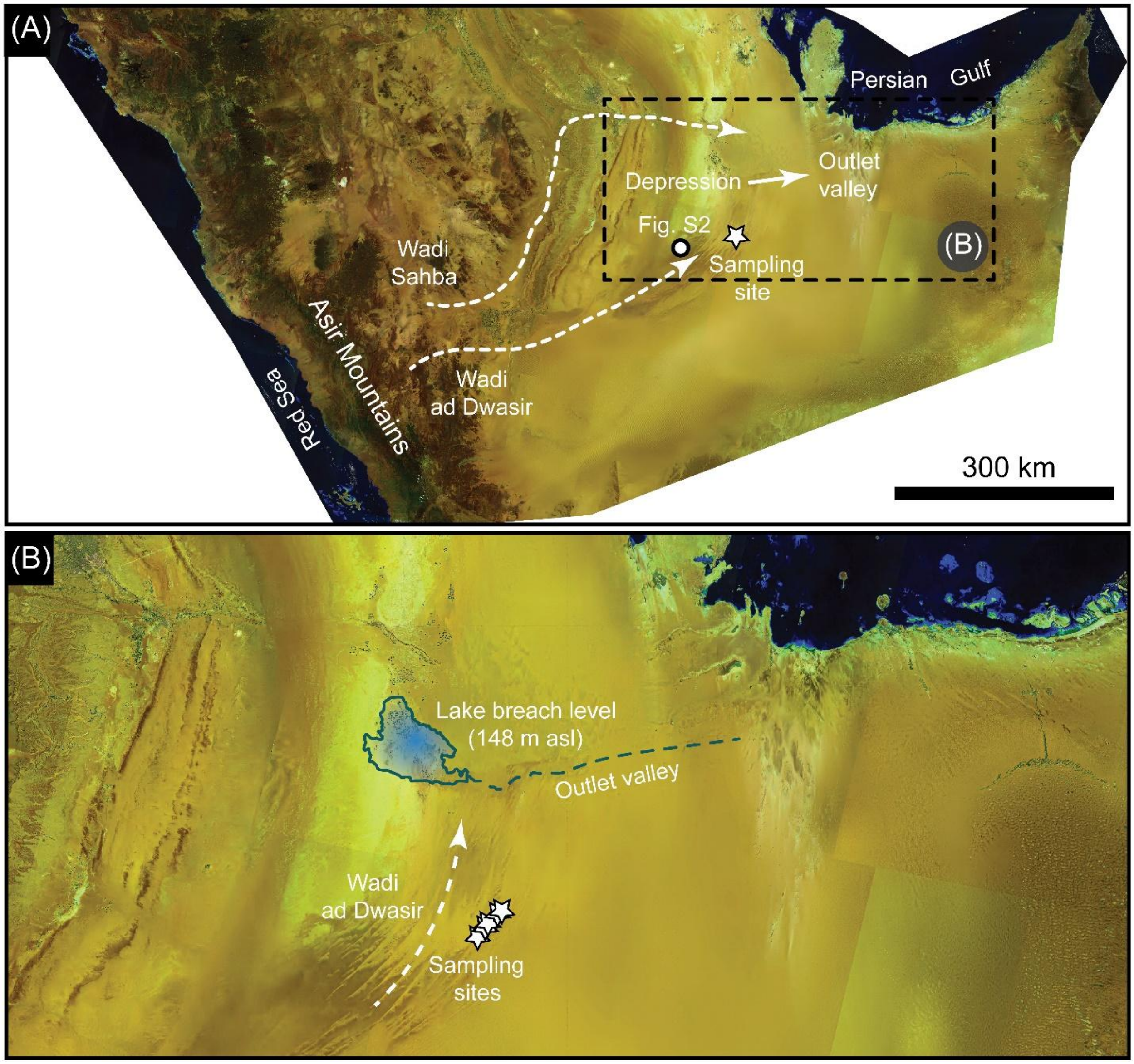

Fig. S1: A mosaic of Landsat satellite imagery displaying the source area of both Wadi ad Dwasir and Wadi Sahba, the breach lake level, as well as the sampling sites.

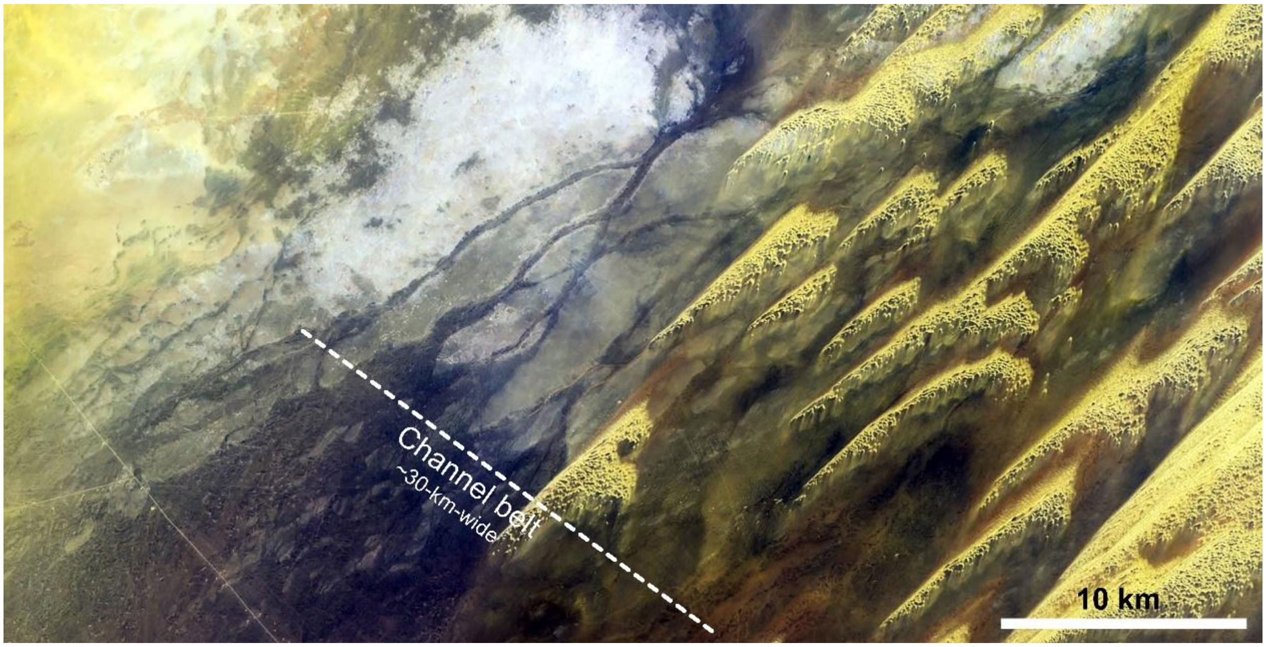

Fig. S2: Landsat 8 images showing a portion of the Wadi ad Dwasir, marking a channel belt within a braided river.

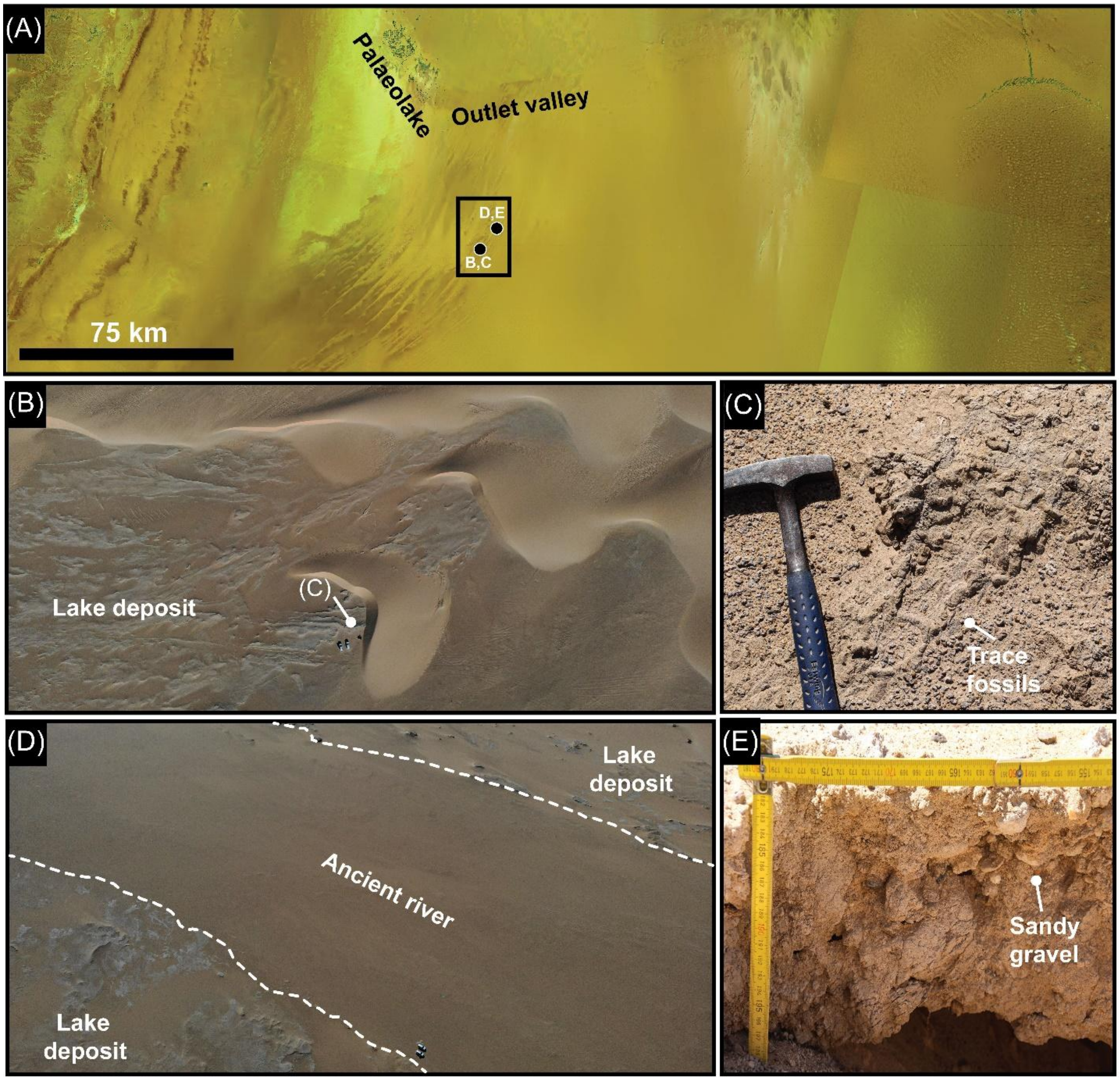

Fig. S3. Overview and Detailed Observations from UAV and Field Photographs. (A) Map illustrating the locations where drone images were captured. (B) and (D) Aerial photographs taken with an unmanned aerial vehicle (UAV), highlighting whitish deposits indicative of lacustrine sediments intersected by ancient river channels (coordinates: 21.935944° N, 49.740503° E for panel B; 22.031781° N, 49.815347° E for panel D). (C) Field photographs of trace fossils located on lacustrine deposits. (E) Close-up of sandy gravels derived from ancient streamflows embedded within the lacustrine deposits.

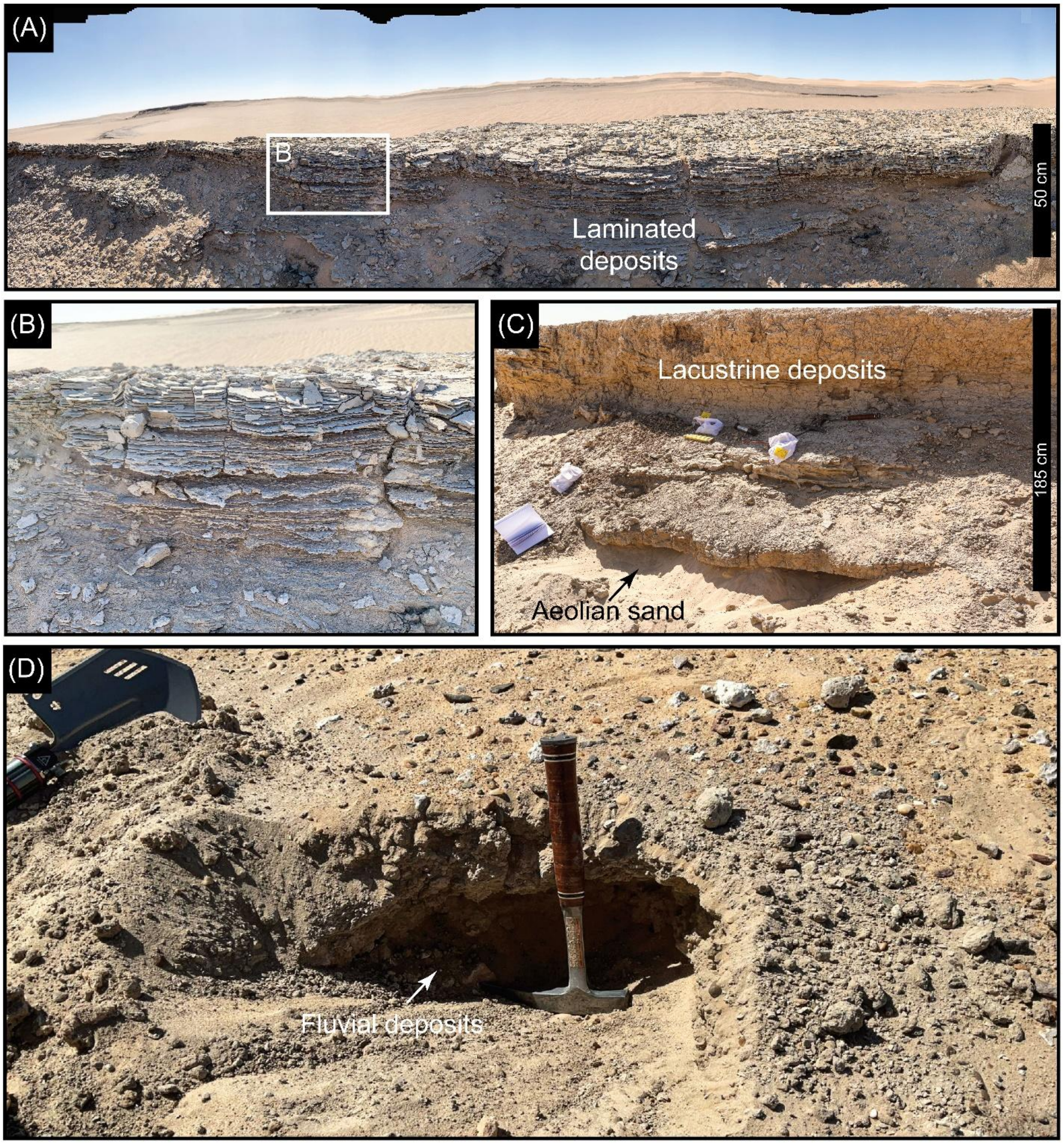

Fig. S4. Field photographs of different sections showing evidence of lacustrine and fluvial deposits. (A) and (B) display approximately 50 cm of undisturbed laminated deposits atop carbonate-rich marl facies (22.059244°N, 49.829894°E). We interpret these features to be either a lacustrine environment or wetland setting. (C) depicts a 1.85 m outcrop of lacustrine deposits overlaying aeolian sands (22.032260°N, 49.813292°E). (D) shows a section of fluvial deposits comprising thin veneers of sandy gravels (21.984818°N, 49.782402°E).

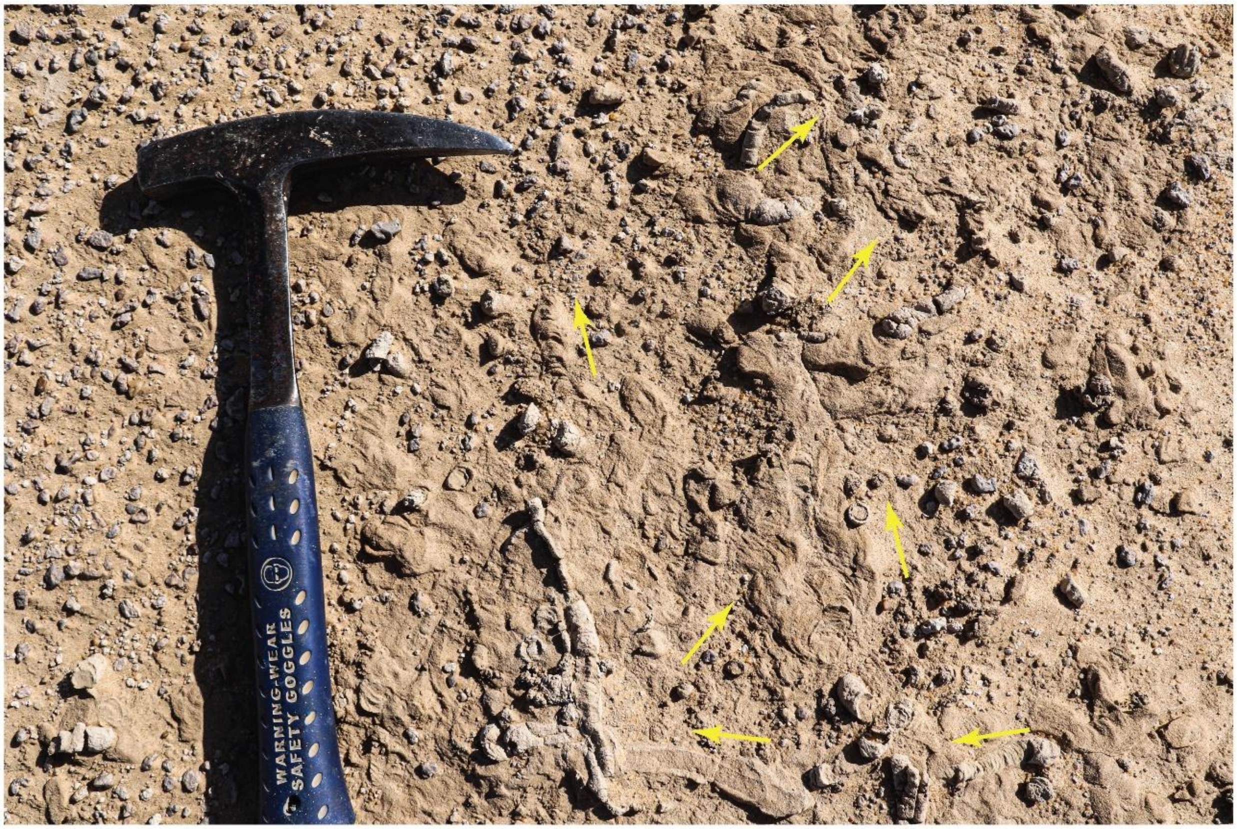

Fig. S5: Trace fossils have been found atop the lacustrine deposits. The features bear a resemblance to Cruziana, Taenidium, and Scoyenia, which suggest that the site was either directly on the shoreline or in its close vicinity, as they preserve the interaction between organisms and sediment at a transitional water-land interface.

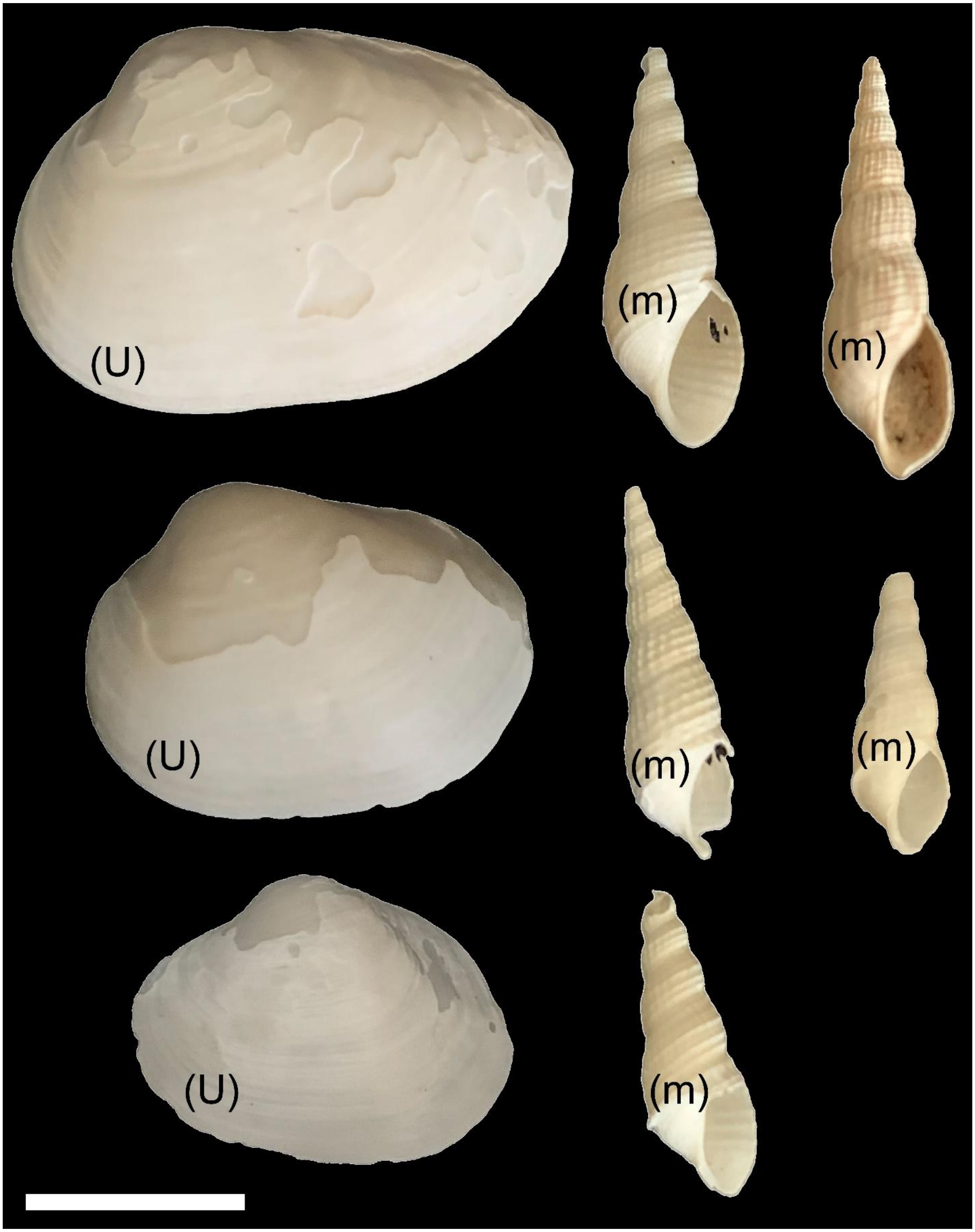

Fig. S6: Various freshwater mollusc fossils (*Unio tigridis "u"* and *Melanoides tuberculatus "m"*) have been found from different lacustrine outcrops, suggesting the presence of a perennial water body. The white scale bar is 15 mm.

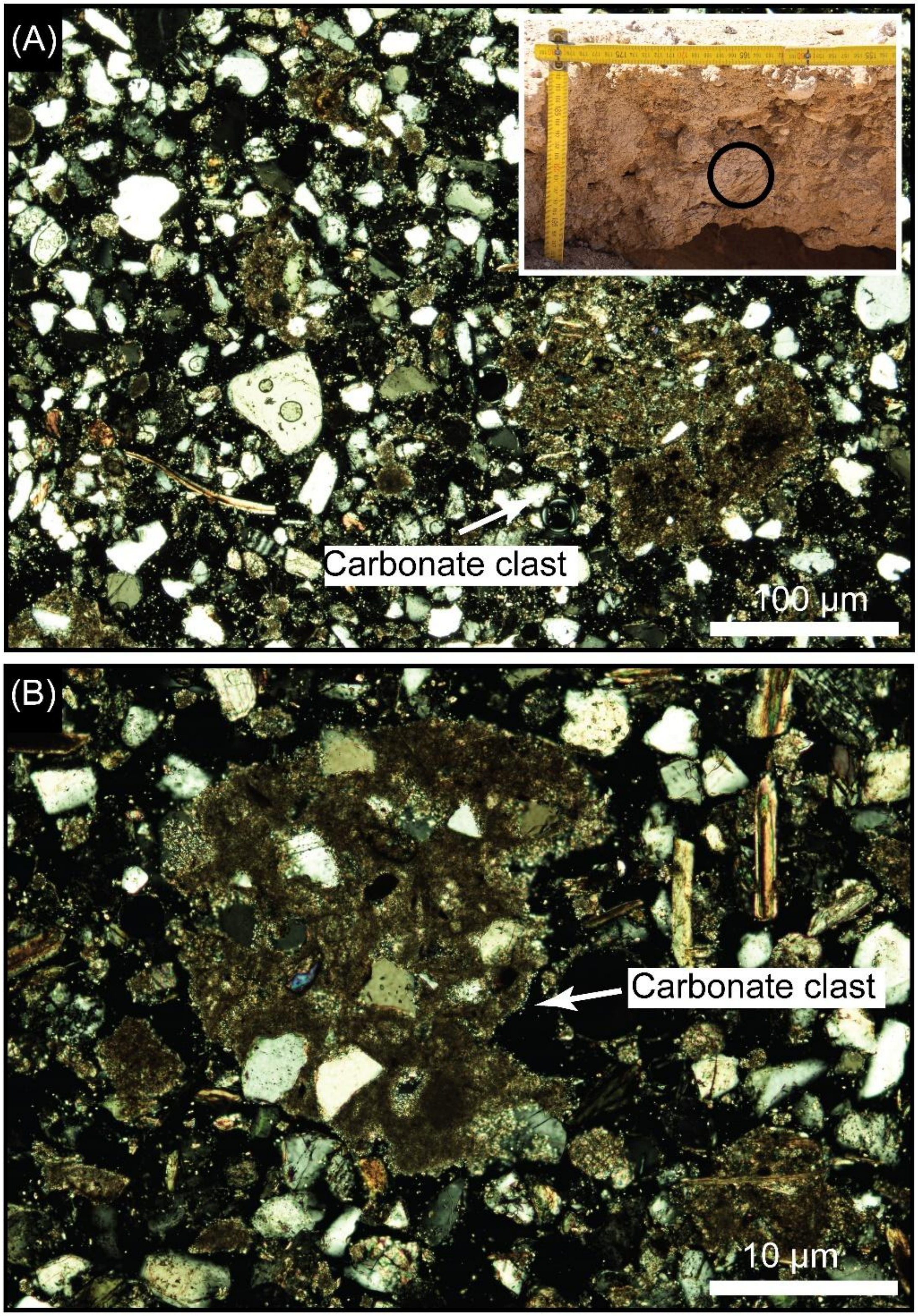

Fig. S7. Photomicrographs depicting carbonate clasts shaped by fluvial processes within a lacustrine environment. The sample was collected from fluvial deposits located at coordinates 21.984765°N, 49.782531°E.

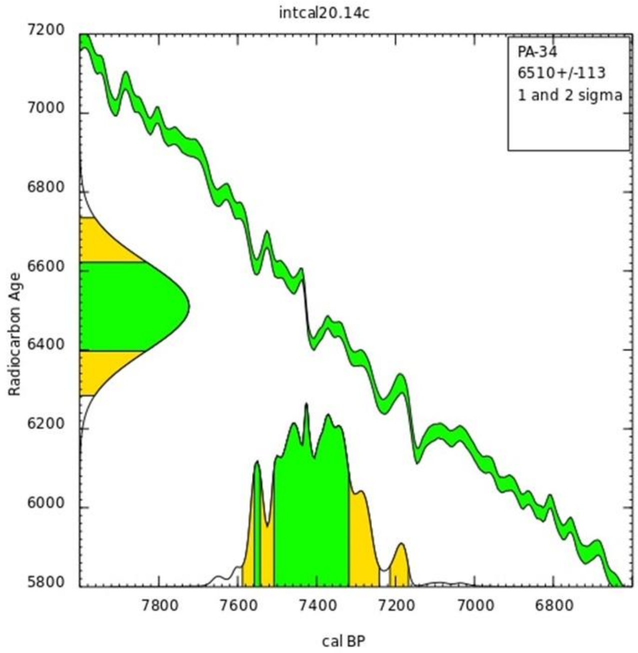

Fig. S8. The uncalibrated age of sample PA-34 in radiocarbon years versus the calibrated age in calendar years before present (cal BP), relative to the IntCal20 calibration curve (Reimer et al., 2020). Calibration was carried out using the Calib 8.20 software (Stuiver and Reimer, 1993).

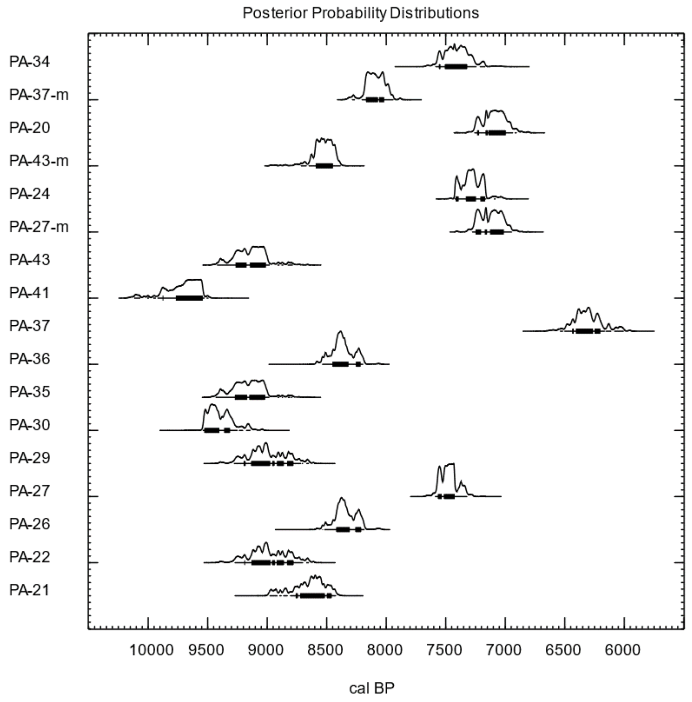

Fig. S9. The calibrated age distributions of all samples in this study in cal BP. Calibration was carried out using the Calib 8.20 software (Stuiver and Reimer, 1993).

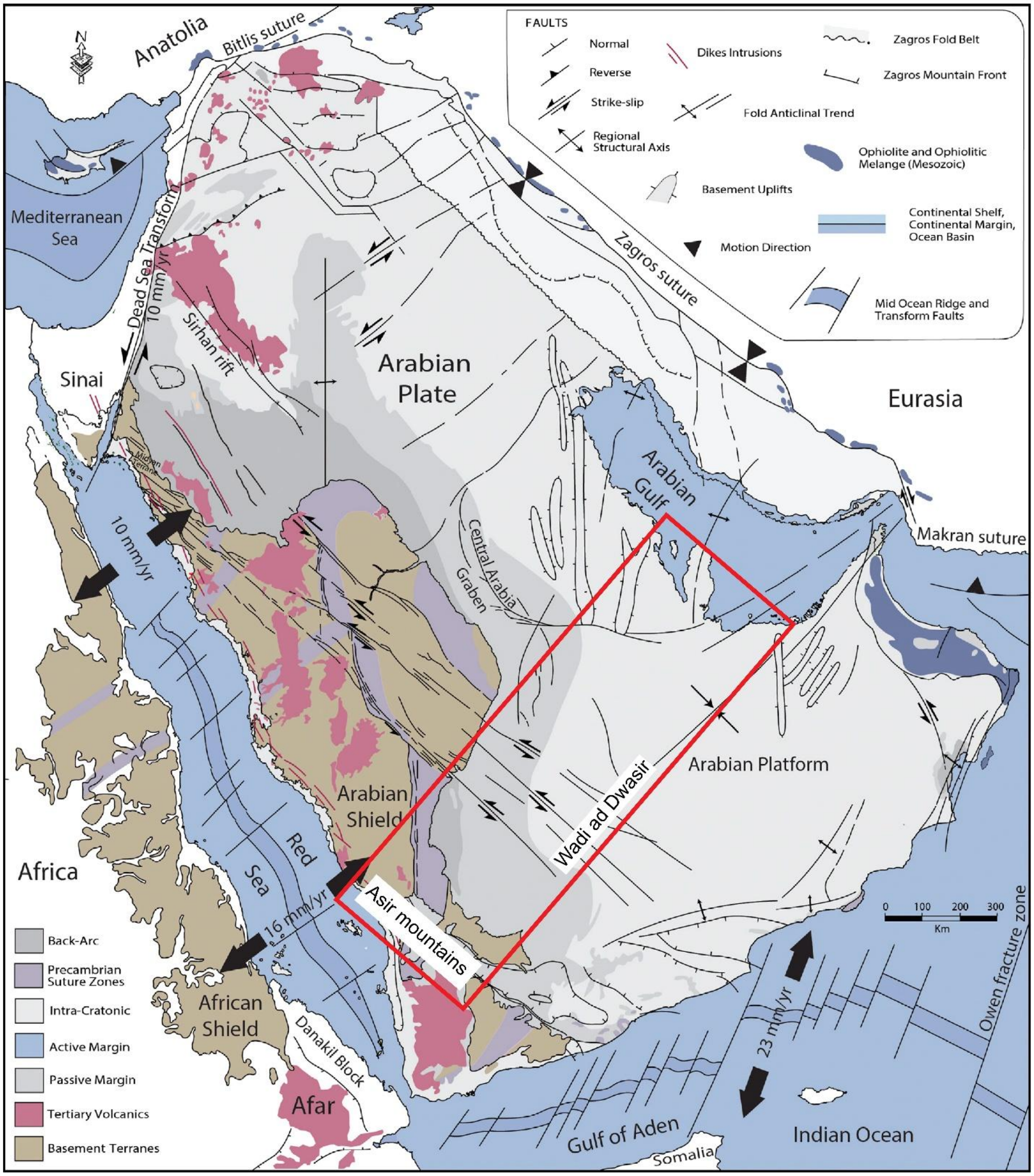

Fig. S10. Geological and tectonic features map of the Arabian Plate and surrounding regions, modified from Aldaajani et al. (2021). The red square highlights the Wadi ad Dawasir region, which includes various tectonic structures within the study area.

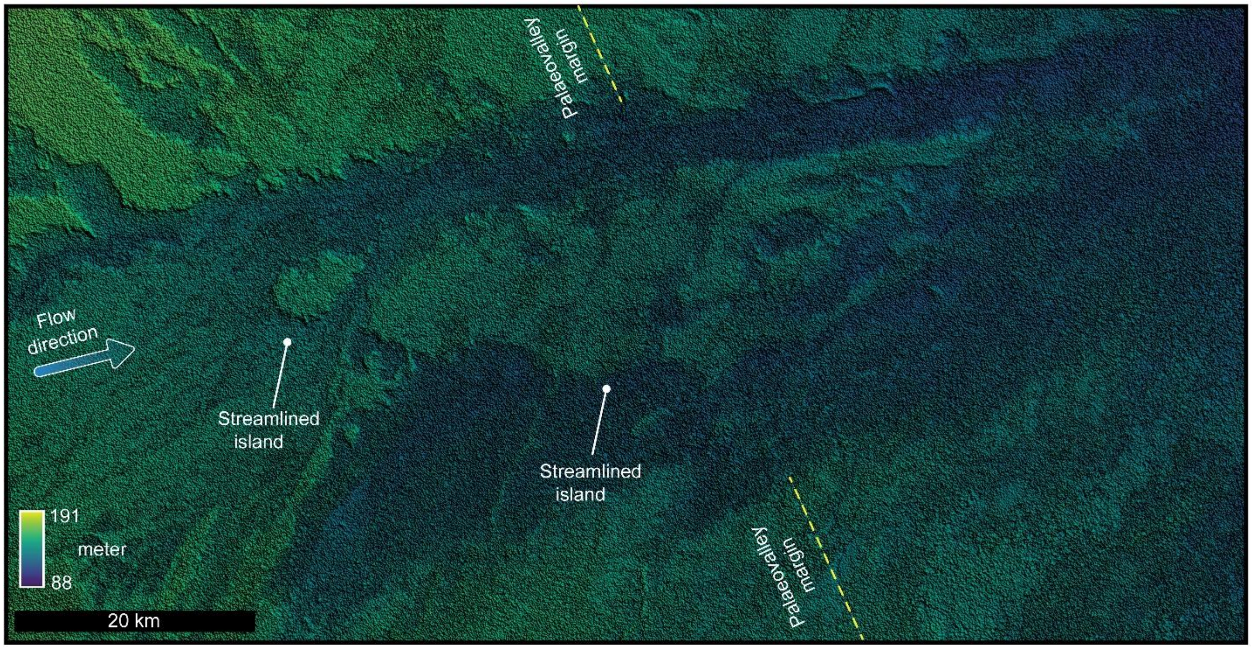

Fig. S11. Three-dimensional view of the palaeovalley showing the valley margin as well as the streamlined islands. Data are derived from a 30-m SRTM digital elevation model.

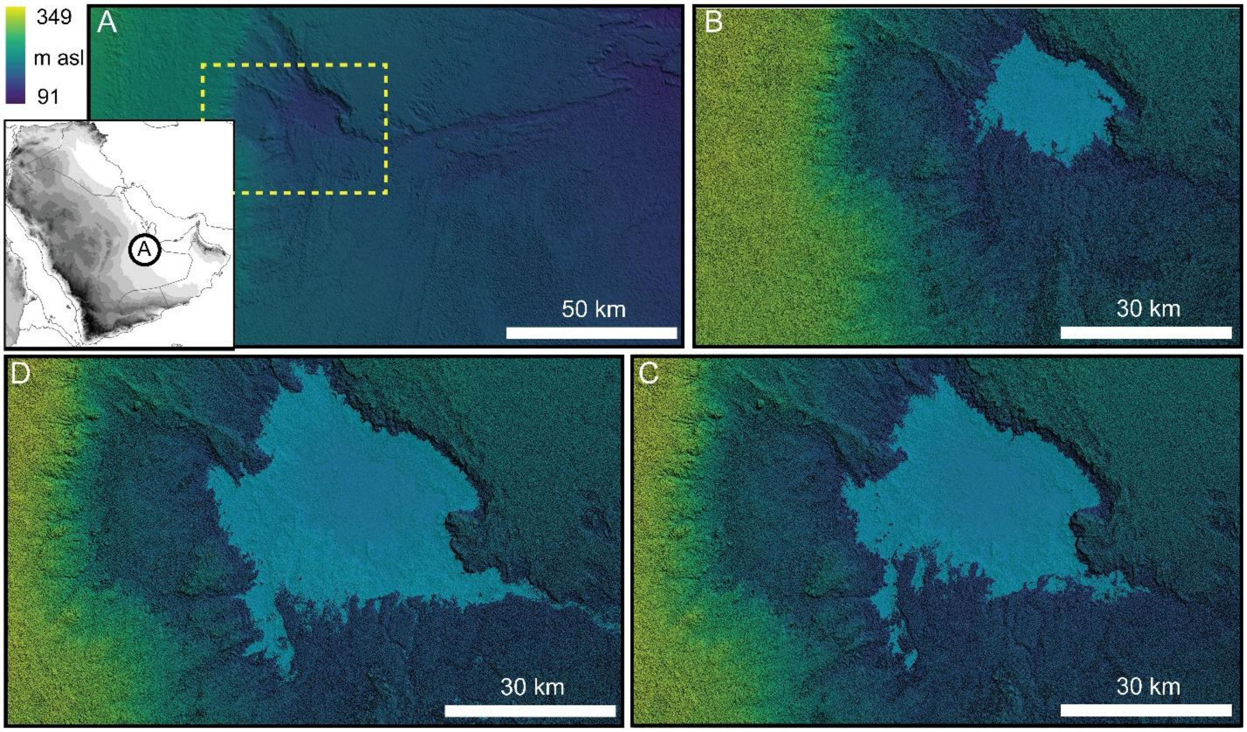

Fig. S12. Modeled water levels illustrate the lake area and the mechanisms of lake filling and breaching at three levels: (A) an overview map displays the regions; (B) at 128 m asl, covering an area of approximately 439 km²; (C) at 141 m asl, with an area of 802 km²; and (D) at 148 m asl, encompassing 1067 km². The background data are derived from a 30-meter-resolution SRTM (Shuttle Radar Topography Mission) digital elevation model.

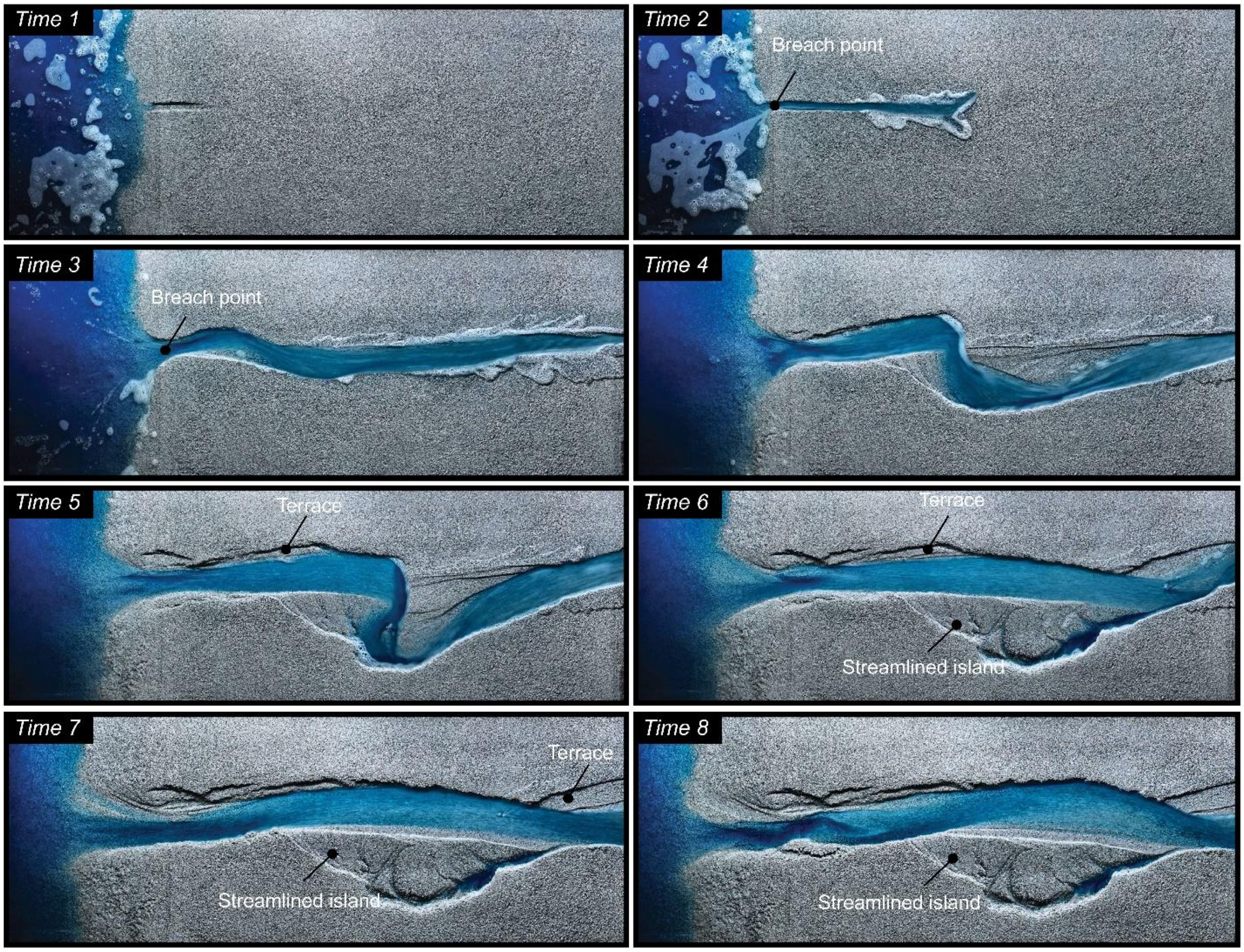

Fig. S13. Sequences of images represent time steps produced from a flume experiment simulating lake overflow, conducted by Marra et al., 2014 (license number: 5759240915535). These images illustrate the process of lake breaching, highlighting that the breach point remains nearly constant throughout the development of the outlet valley. Additionally, the images show the morphologies associated with the lake breach, including terraces and a streamlined island.

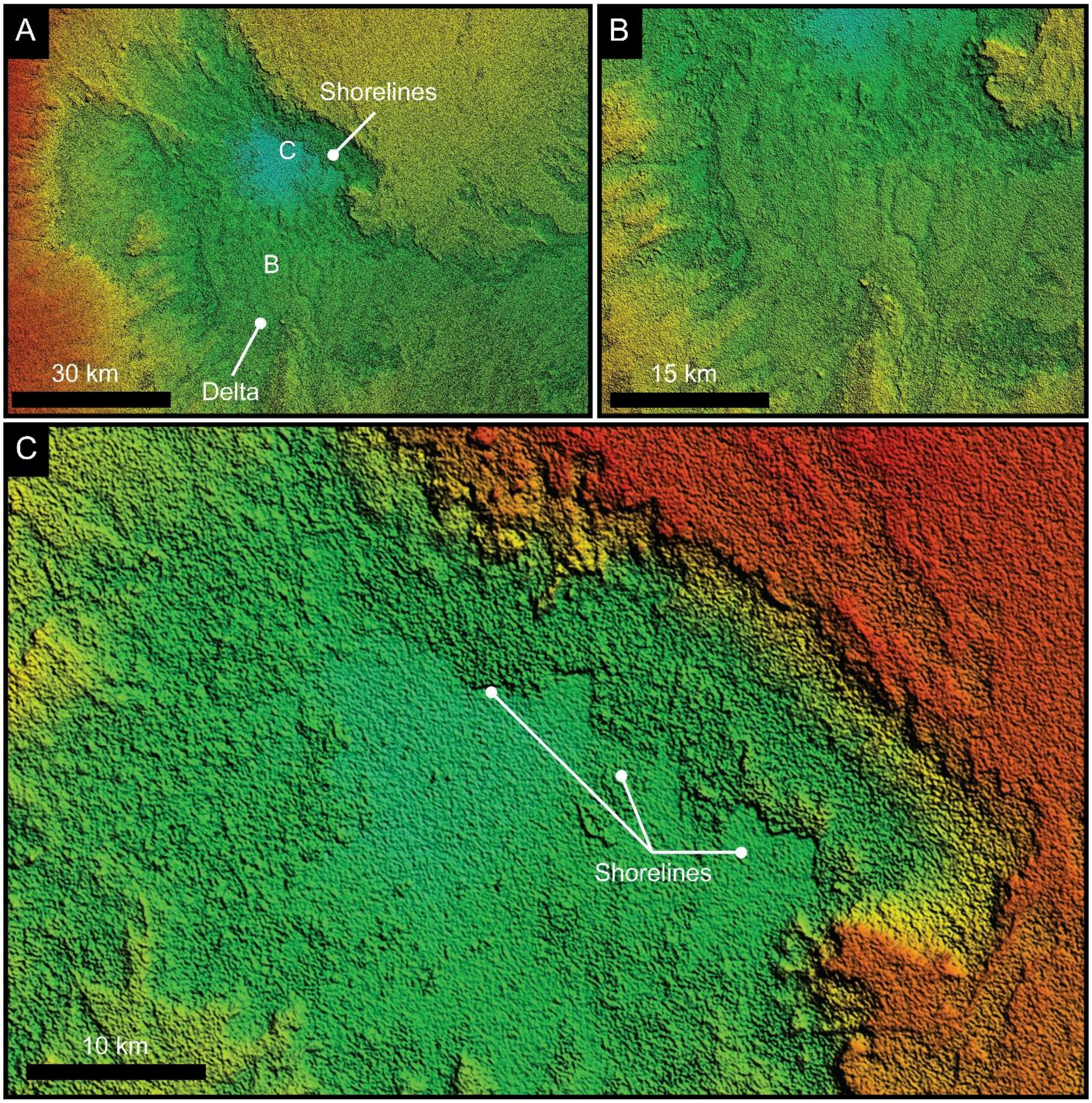

Fig. S14. (A) A digital elevation model derived from 30-m SRTM data, illustrating the depression bounded by a delta and a shoreline. Panels B and C show close-up views of both the delta and shoreline morphologies.

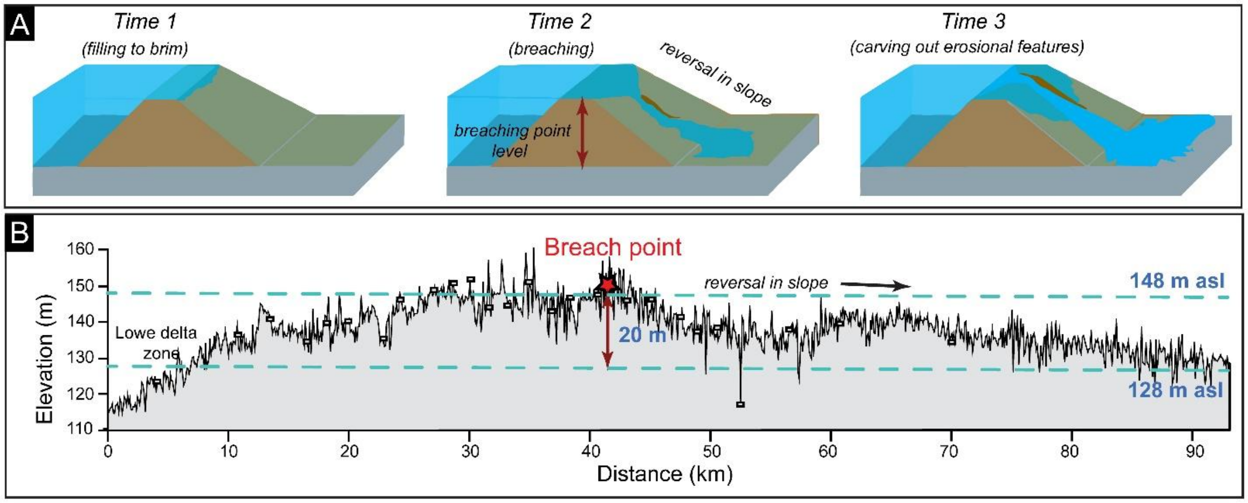

Fig. S15. A conceptual diagram illustrating the overtopping breach mechanism, modified from Flynn et al., 2021. Panel B presents a longitudinal profile of the outlet valley, demonstrating the elevation changes over space in alignment with the overtopping breach model. It also shows how and where the breach point level can be measured.

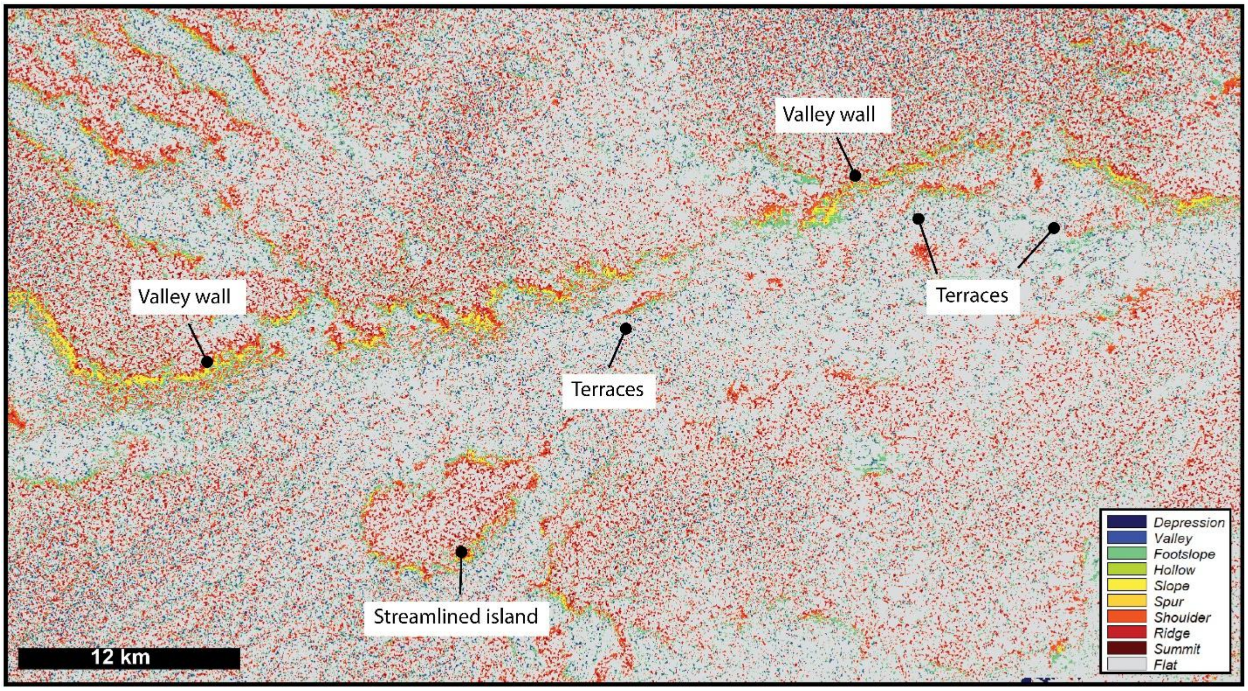

Fig. S16. Automated terrain classification by the Geomorphons algorithm that detects both the high slopes associated with the valley walls and the flat surfaces likely representing preserved terraces. The presence of terrace-like forms suggests the presence of episodic formation.

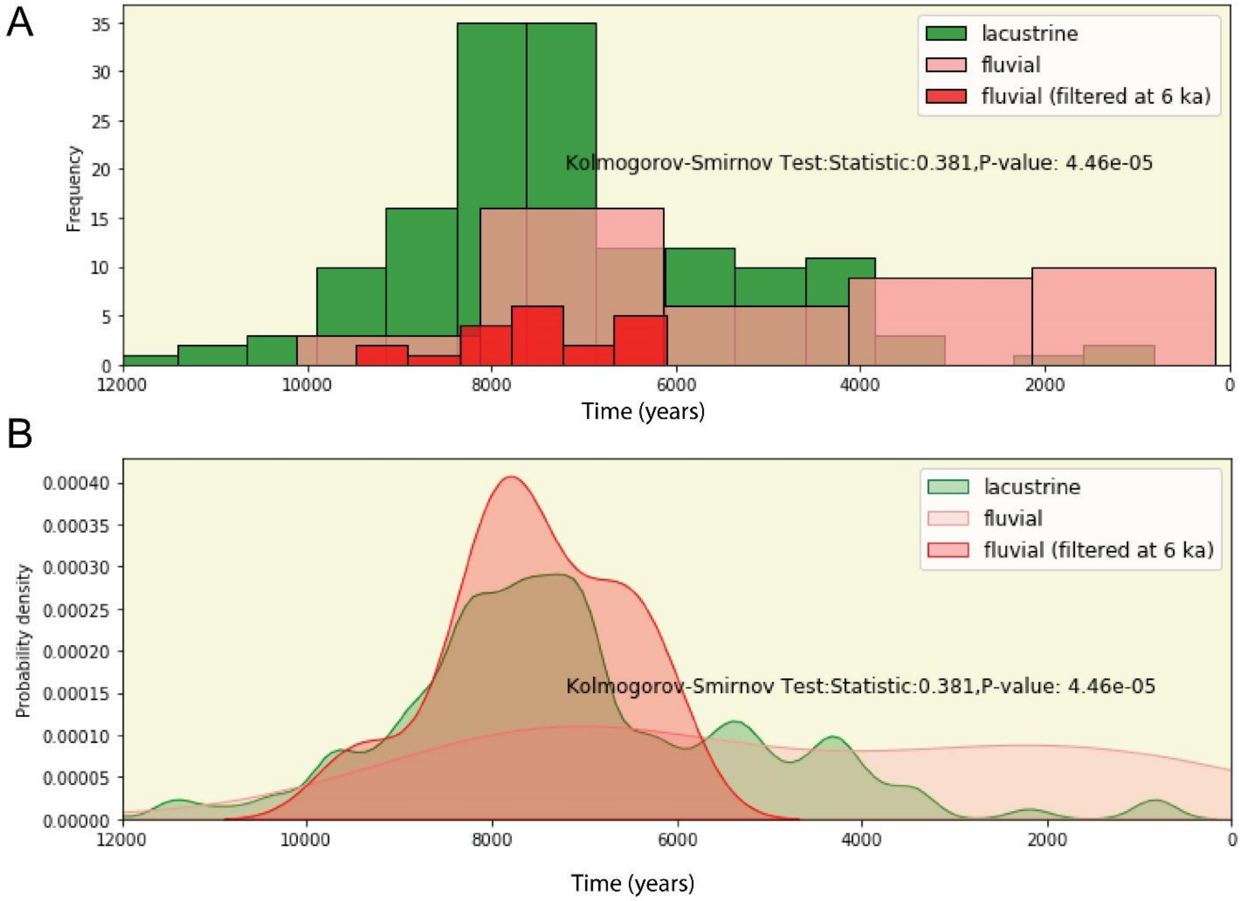

Fig. S17: (A) and (B) histograms showing the distribution of the fluvial and lacustrine ages over the past 12,000 years. The Kolmogorov-Smirnov test results indicate a significant statistical difference between the age clusters of fluvial and lacustrine environments.

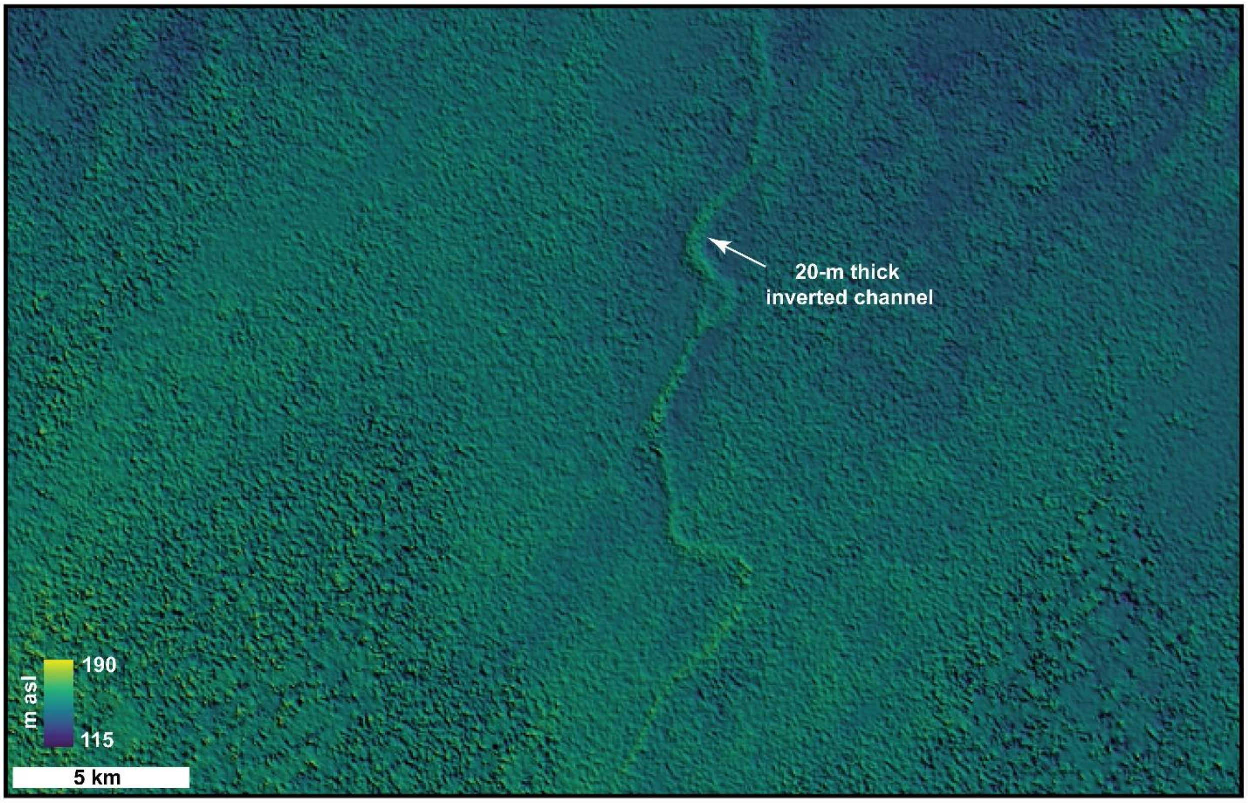

Fig. S18. Digital elevation model showing a 20-m-thick inverted channel, demonstrating that significant erosion occurred since the middle Holocene. The figure was derived from 30-m SRTM data. (22° 45´ 07.73" N 50° 10´ 08.85" E).

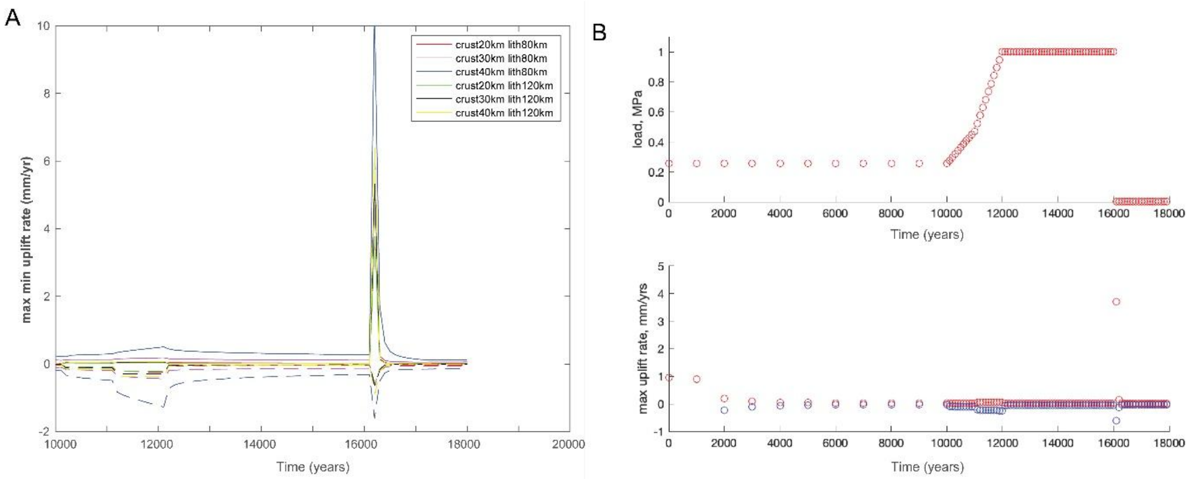

Fig. S19. (A) and (B) Viscoelastic deformation model shows the region's load and minimum and maximum uplift and subsidence rates over the past 18,000 years.

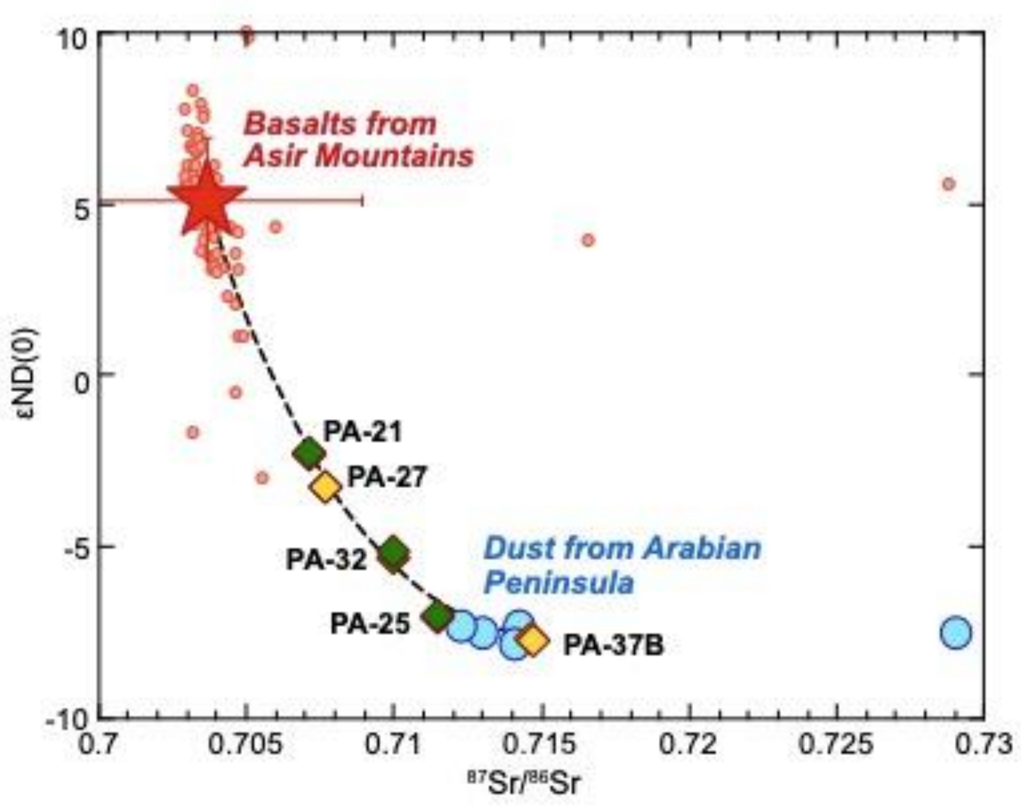

Fig. S20. Scatter plot of $^{87}Sr/^{86}Sr$ versus εNd showing a mixing curve between radiogenic volcanic sources and unradiogenic crystalline present-day dust. Red points are individual whole rock analyses obtained from the GEOROC database (https://georoc.eu/georoc/new-start.asp, accessed on November 7, 2023) and median (red star) with standard deviation. Blue points are offshore and onshore dust samples from the Arabian Peninsula. Green diamonds are lacustrine samples and yellow diamonds are fluvial samples. Two samples (PA-25 and PA-37B) are almost entirely composed of local crystalline material, such as dust while the other samples are composed of a mixture of remote volcanic material and local crystalline material.

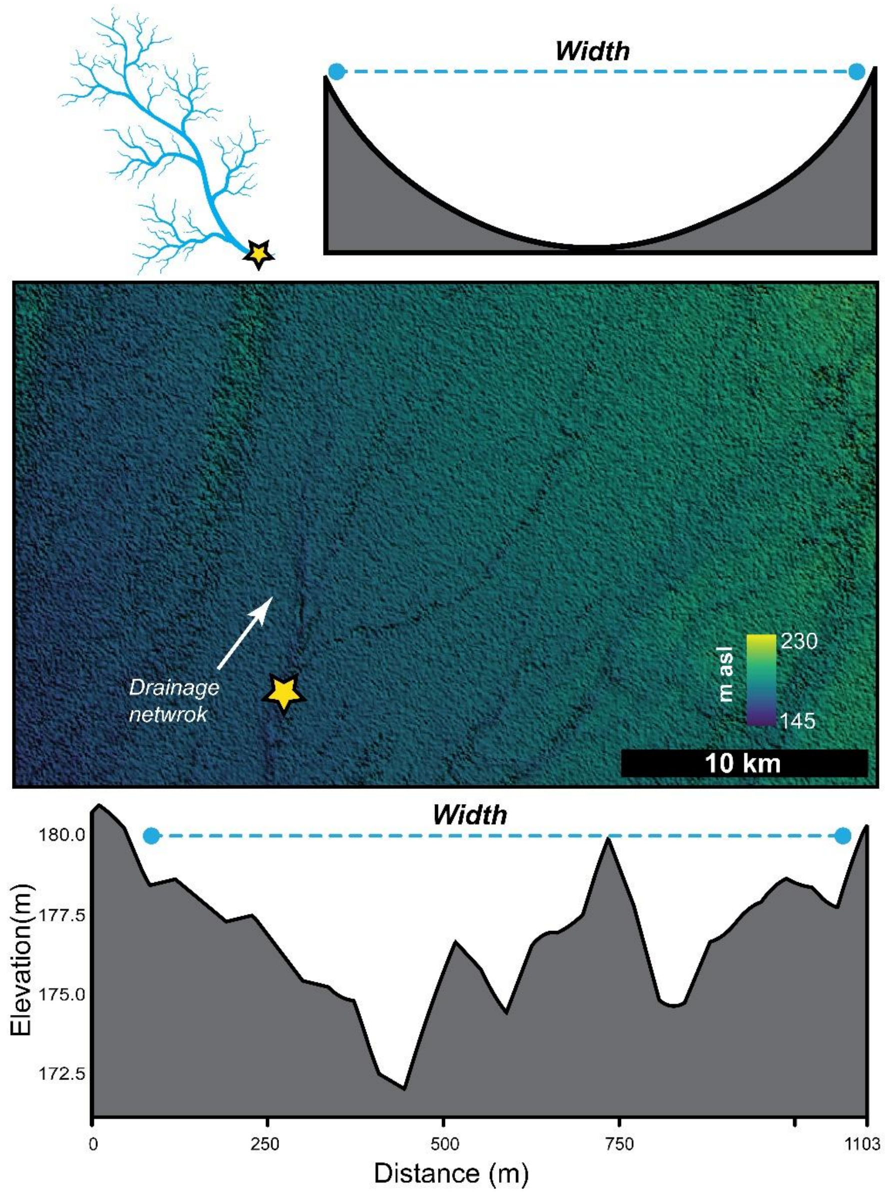

Fig. S21. A sketch illustrating how the width was measured along the drainage basin to estimate the palaeodischarge. The figure also shows how the width measurements were obtained from SRTM data.

Table legends

Supplementary data 1 and 2. A compilation of ages from morphological features indicating hydrological changes: (1) optical luminescence ages, and (2) radiocarbon ages.

Supplementary data 3. Results of dated samples collected from both fluvial and lacustrine environments.

Supplementary data 4. Results of neodymium (Nd) and strontium (Sr) isotope analyses measured on the siliciclastic fraction of the sediments.

Supplementary references

1. Stuiver, M. & Reimer, P. J. Extended 14C data base and revised CALIB 3.0 14C Age Calibration Program. *Radiocarbon* **35,** 215–230 (1993).
2. Reimer, P. J. *et al.* The intcal20 Northern Hemisphere radiocarbon age calibration curve (0–55 cal KBP). *Radiocarbon* **62,** 725–757 (2020).
3. Aldaajani, T. Z., Almalki, K. A. & Betts, P. G. Plume versus slab-pull: Example from the Arabian Plate. *Frontiers in Earth Science* **9,** (2021).
4. Flynn, S., Zamanian, S., Vahedifard, F., Shafieezadeh, A. & Schaaf, D. Data-driven model for estimating the probability of riverine levee breach due to overtopping. *Journal of Geotechnical and Geoenvironmental Engineering* **148,** (2022).
5. Marra, W. A., Braat, L., Baar, A. W. & Kleinhans, M. G. Valley Formation by groundwater seepage, pressurized groundwater outbursts and crater-lake overflow in flume experiments with implications for Mars. *Icarus* **232,** 97–117 (2014).
