# Supplementary material for: Monsoonal imprint on late Quaternary landscapes of the Rub’ al Khali Desert
Source: Commun Earth Environ. 2025 Apr 3;6(1):255. doi: 10.1038/s43247-025-02224-1 (PMC11968403; doi:10.1038/s43247-025-02224-1)
Supplement: Supplementary file 3 — Description of Additional Supplementary Files [file 43247_2025_2224_MOESM3_ESM.pdf]

Description of Additional Supplementary Files

File name: Supplementary Movie S1

Movie S1 illustrates how precipitation patterns have changed over the 5 past ~24,000 years across the Saharo-Arabian Desert.

File name: Supplementary Data 1, 2, 3, and 4

Supplementary data 1 and 2. A compilation of ages from morphological features indicating hydrological changes: (1) optical luminescence ages, and (2) radiocarbon ages.

Supplementary data 3. Results of dated samples collected from both fluvial and lacustrine environments.

Supplementary data 4. Results of neodymium (Nd) and strontium (Sr) isotope analyses measured on the siliciclastic fraction of the sediments.
